# Prevalence and determinants of hypertension in a semi-urban population: a cross-sectional study in Dschang (West Region of Cameroon)

**DOI:** 10.11604/pamj.2024.48.157.43781

**Published:** 2024-08-06

**Authors:** Sylvain Raoul Simeni Njonnou, Cédric Fritz Gerald Eyenga Bangbang, Christian Ngongang Ouankou, Fernando Kemta Lekpa, Christian Deube Ngako, Liliane Mfeukeu-Kuate, Marie-Josiane Ntsama Essomba, Hamadama Abdoul Salam, Herna Stella Chimy Tchounchui, Clarisse Mapa-Tassou, Aimée Tiodoung Timnou, Siméon Pierre Choukem

**Affiliations:** 1 Department of Internal Medicine and Specialties, Faculty of Medicine and Pharmaceutical Sciences, University of Dschang, Dschang, Cameroon,; 2 Dschang Regional Hospital Annex, Dschang, Cameroon,; 3 Department of Public Health, Faculty of Medicine and Pharmaceutical Sciences, University of Dschang, Dschang, Cameroon,; 4 Yaoundé Teaching Hospital, Yaoundé, Cameroon,; 5 Douala General Hospital, Douala, Cameroon,; 6 Department of Internal Medicine and Specialties, Faculty of Medicine and Biomedical Sciences, University of Yaoundé I, Yaoundé, Cameroon,; 7 Yaoundé Central Hospital, Yaoundé, Cameroon,; 8 Figuil District Hospital, Figuil, Cameroon,; 9 Ministry of Public Health, Yaoundé, Cameroon,; 10 Health and Human Development (2HD) Research Network, Douala, Cameroon

**Keywords:** Hypertension, prevalence, semi-urban area, Dschang

## Abstract

**Introduction:**

hypertension is a major public health problem worldwide, associated with considerable morbidity and mortality. Although the national prevalence of hypertension is well established, its prevalence in semi-urban areas is poorly known. This study aimed to establish the prevalence and determinants of hypertension in a semi-urban area.

**Methods:**

we conducted a cross-sectional community study in the adult population of the Dschang Health District from February to May 2022. Consent to participate was obtained and data was collected through a face-to-face interview using a predesigned questionnaire. Collected variables included sociodemographic characteristics, previous education on weight loss, salt, alcohol, and tobacco consumption, and blood pressure level after 45 min of rest.

**Results:**

we recruited 706 participants with a mean age of 53.11 years. The prevalence of hypertension was 57.6%, with 13.02% for grade 1; 64.87% for grade 2 hypertension, and 22.11% for grade 3. The determinants significantly associated with hypertension in multivariate analysis were: age over 40 years (aOR: 3.23, 95% CI 1.91 - 5.47; p<0.001), being a civil servant (aOR: 4.82, 95% CI 2.44 - 9.54; p<0.001), being unemployed (aOR: 2.95, 95% CI 1.32 - 6.57; p =0.008), tobacco consumption (aOR: 1.93, 95% CI 1.49 - 2.38; p <0.001), hypertension among siblings (aOR: 4.56, 95% CI 1.30 - 15.95; p =0.017), diabetes mellitus (aOR: 3.09, 95% CI 1.61 - 5.94; p <0.001), obesity (aOR: 2.8, 95 CI 1.10 - 7.74; p<0.001), previous therapeutic education (over salt consumption: [aOR: 1.7 95% CI 1.08 - 2.67; p<0.001], alcohol consumption: [aOR: 2.12, 95% CI 1.24 - 3.61; p<0.04] and weight loss [aOR: 2.51, 95% CI 1.76 - 3.57; p<0.001]), presence of palpitations (aOR: 22.63, 95% CI 1.83 - 278.6; p=0.015) and reduced vision (aOR: 1.97, 95% CI 1.08 - 3.6; p=0.026).

**Conclusion:**

a high prevalence of hypertension was found in the Dschang Health District. Sociodemographic characteristics, family and personal history and some clinical manifestations were associated with hypertension. There is a need to implement hypertension prevention strategies in semi-urban settings.

## Introduction

Hypertension is the main risk factor for cardiovascular disease. It represents today a major public health problem, on a global scale because of its frequency and the risks of cardiovascular and renal diseases which are attached to it, but also because of the expenses or disabilities it generates. It is unevenly distributed across continents and countries. Its prevalence is estimated at 40% and 15.2% respectively in the United States and France [1]. It affects all races, all ethnic groups, and all socio-professional strata [2] and today constitutes a major challenge not only in the general population but also in the workplace because of the risk of disability linked to this condition. In the African region, 20 million people are said to be affected [3].

This estimated prevalence also varies by country in sub-Saharan Africa: 15% for Algeria, 30% for Mauritius and Seychelles, 20-35% for Gabon, and 9.5% for Gambia [4]. In most West African countries, there is an urban/rural gradient, with a very high prevalence in urban areas but increasing in semi-urban or rural areas [5]. Similarly, the prevalence in the Ivory Coast was superposable to the other countries: during the May Month Measurement in 2017, a prevalence of 20.4% was found [6].

In Cameroon, the prevalence is increasing in the general population, going from 20.8% in 2007 to 24.2% in 2012 to 29.7% in 2015 [7,8]. A study carried out in 2019 in Edéa found a prevalence of hypertension in the workplace at 14% [9]. However, recent data have shown a prevalence above 30% of hypertension in the general population [10]. There is, therefore, a regional disparity between rural and urban areas, with the prevalence of hypertension in rural areas lower than in urban areas but increasing over time [11-13]. This heavy burden can have negative consequences as a large proportion of hypertensive subjects may remain undiagnosed, untreated, or poorly managed, posing a high risk of morbidity and mortality due to potentially preventable complications of hypertension such as stroke and heart disease [8,14], hence the importance of our research in semi-urban areas. This study aimed to establish the prevalence and the determinants of hypertension in a semi-urban area.

## Methods

**Study design and setting:** this was a cross-sectional study that took place in the Health District of Dschang from December 2021 to May 2022 in the city of Dschang, targeting adult patients living in the Dschang Health District. This study aimed to determine the prevalence of hypertension and identify its determinants among community dwellers of Dschang. The Health District of Dschang is located in the Menoua subdivision, in the West Region of Cameroon. It extends over an area of 262 km^2^ distributed in its urban area which has 20 communities and in the rural area which has 96. The five groups that make it up are Foto: 99 km^2^; Foréké-Dschang: 86 km^2^; Fongo-Ndeng: 31 km^2^; Fossong Wentcheng: 18 km^2^; Fotetsa: 11 km^2^; Urban center: 7 km^2^. The city of Dschang is a 45-minute drive from Bafoussam (60 Km), four hours from Douala (300 Km), and five hours from Yaoundé (400 Km). The surface area of the urban space of Dschang is estimated at 5655 ha and is located in the intercession of the territory of the Foto and Foréké-Dschang chiefdoms. The Commune of Dschang is limited to the north by the Commune of Nkong-Zem; to the south by the Municipality of Santchou; to the west by the Municipality of Fongo-Tongo; to the east by the Municipality of Fokoué; to the south-west by the Municipality of Fontem [15].

**Study population:** this study targeted the adult (over 21 years) population from both sexes of Dschang, living there for more than three months. We first randomly selected 3 health areas among all the health areas in the city of Dschang. In each health area, we randomly selected 50% of all quarters, in which 50% of the blocks have been randomly selected. In the selected blocks, we investigated all the households in which the people eligible and wishing to participate in our study were present. All people living in the selected health areas, quarters, and blocks being at home at our passage and wishing to participate in the study were included. Participants who had not stayed 15 days in the month in the city or living in the health area for less than 3 months were excluded. Informed consent was obtained from all study participants and the study protocol was implemented according to the recommendations of the latest revision of the Helsinki Declaration [16]. The sample size was calculated using Lorentz’s formula (Stat Calc of EPI Info® Software). Using the national prevalence of 29.7% in Cameroon (according to national 2018 data), with an 80% power to detect significant associations or differences, and a 5% accepted margin of error. This gave us a minimum sample size of 461 participants using a margin of error of 5% (standard value of 0.05). The health areas in which the recruitment was to take place were randomly drawn and 3 of them were selected. In each health area, we randomly selected 50% of all quarters, in which 50% of the blocks have been randomly selected. In the selected blocks, we investigated all the households in which the people eligible and wishing to participate in our study were present. Participants were selected consecutively.

**Data collection:** data were collected from the participant alone or, if necessary, in the presence of an interpreter (with the participant's consent) to facilitate communication. Participants were provided with an information leaflet explaining the aims of the study, and additional explanations were given if requested. Information was collected only after a positive response from the participant, who was given a consent form to sign. Data were collected using a pre-tested, standardized, anonymized questionnaire designed for this purpose. The variables were the following: i) socio-demographic characteristics (gender, age, marital status, level of education, activity, health status, area of residence); ii) chronic diseases and comorbidities: cardiovascular risk factors (known hypertension, obesity, sedentary, diabetes, gout, chronic kidney disease), history of cardiovascular events (stroke, cardiac heart failure, chronic kidney disease), alcohol and tobacco consumption; iii) clinical variables (blood pressure [BP] level taken with an OMRON MX2 Basic® device following the 11-BP steps, weight, height, waist circumference, and symptoms that can be associated with increased BP or its complications such as asthenia, headaches, dyspnea, reduced vision, palpitations) [17]. Given the fact that 30-minute office blood pressure (30-OBP) was found in previous studies superposable to ambulatory blood pressure monitoring (ABPM) and a local study found more accurate the 45-OBP in participants living with diabetes and hypertension, we considered hypertensive participants with BP ≥ 140/90 mmHg after 45 min of rest [18-20].

**Operational definitions:** i) hypertension: systolic BP ≥ 140 mmHg and/or diastolic BP ≥ 90 mmHg defined hypertension at 45 min of rest. People with previous diagnoses or treatment for hypertension were also considered hypertensive; ii) obesity was defined as a body mass index (BMI) of ≥ 30 kg/m^2^, and overweight was defined as a BMI between 25 and 29.9; iii) diabetes: the participant was declared diabetic according to the following criteria defined by the American Diabetes Association: fasting plasma glucose (FPG) level ≥ 126 mg/dL (7.0 mmol/L) on two separate occasions, random blood glucose ≥ 200 mg/dL (11.1 mmol/L), two hours plasma glucose concentration ≥ 200 mg/dL (11.1 mmol/L) after 75g anhydrous glucose in an oral glucose tolerance test (OGTT) [21]. People with a previous diagnosis or treatment for diabetes were also considered for enrolment; iv) sedentary lifestyle: was defined as the absence of any physical activity (absence of at least 3 walking episodes of 45 min in a week); v) waist circumference > 94 cm in men or 80 cm in women was high; vi) excessive alcohol consumption was based on intake of either more than three (two for women) standard glasses of wine per day or more than ten (five for women) local beers per week. Traditional alcoholic beverage consumption was not assessed [18]; vii) current smoking was defined as the consumption of at least one cigarette per day; viii) a monthly income of less than 86.3 $US defined as a low-social class. Other social classes were classified into the middle (by income between 86.3 and 258.9 $US) and the high (by an income above this amount) [22].

**Statistical analysis:** the data was collected by the online software KOBO COLLECT® using an Excel database designed beforehand. Once collected, the database was extracted and analyzed by Epi info® software version 7.2.2.6 (Atlanta, GA, USA) and STATA® version 16 (College Station, TX, USA). Qualitative variables were represented in frequencies and percentages by bar and sector tables and diagrams and quantitative variables were expressed by means and standard deviations. Chi-squares were used to compare variables. Bivariate analyses were performed and variables with significant p-values (less than 5%) were entered inside a logistic regression to identify the factors associated with the presence of hypertension among participants.

**Ethical considerations:** this work was approved by the Institutional Council of the Faculty of Medicine and Pharmaceutical Sciences, University of Dschang, Cameroon, and received an ethical clearance (Ethical Clearance from the Regional Committee of Ethics and Research for West N°2022/06/037/CE/CRESH-OU/VP) and Obtaining Research Authorization from the Head of the Dchang Health District. We conducted this study in strict compliance with the fundamental principles of scientific research in medicine. Participants were free to participate in the study without external constraints. We obtained an informed and signed consent form for each participant.

## Results

**Figure 1:**
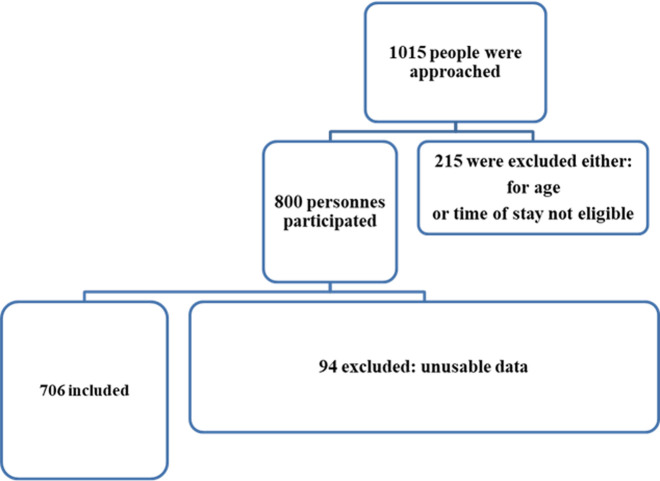
participants flow chart

**Figure 2:**
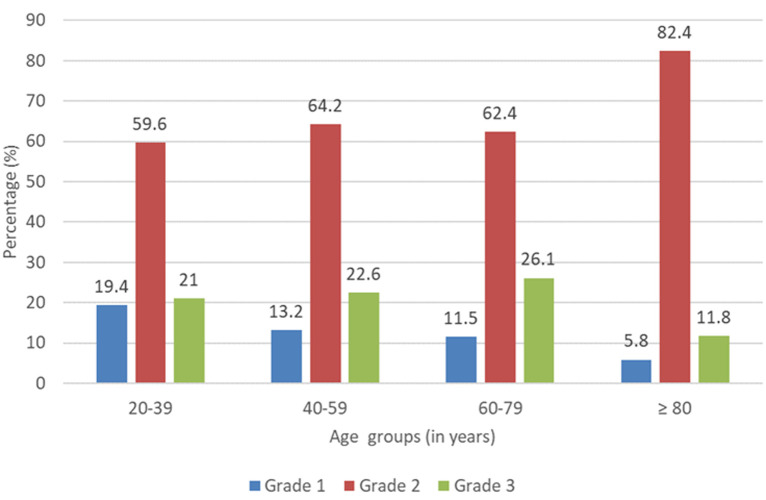
participant’s classification by age group and hypertension grade

**Participants:** a total of 706 participants (261 men) were selected with a mean age of 53.11 ± 17.08 years (Figure 1). People aged 60 and over accounted for more than half of the study population (Figure 2). People with a university level accounted for a third of the study population, while out-of-school participants represented just over a third of the population. The majority of our participants were employees while 16% of them were traders and 15% students. A quarter of participants had a mother (24%) and siblings (25%) living with hypertension; only 15% of participants had a father with hypertension. More than three-quarters (76.96%) of the participants had a comorbidity (alcohol consumption and diabetes being the most common comorbidities). Only 20.7% of participants were already aware of having hypertension. The most common complaints among our participants were in decreasing order: asthenia (27%) followed by headache (15.3%) decreased visual acuity (14.9%), and palpitations (12.5%). Table 1, Table 2, and Table 3 present the characteristics of the study population.

**Table 1:** sociodemographic characteristics of the study population

Variables	Terms	Number (n)	Percentage (%)	CI (95%)	
**Age group (years)**	<40	177	25.07	22.01	28.40
	40 - <50	107	15.16	12.70	17.99
	50 - <60	131	18.56	15.86	21.59
	60 - <70	155	21.95	19.06	25.16
	70 - <80	106	15.01	12.57	17.84
	80 - <90	27	3.82	2.64	5.51
	90 - <100	3	0.42	0.14	1.24
**Sex**	Female	445	63.03	59.41	66.51
	Male	261	36.97	33.49	40.59
**Health areas**	Fiala Foreke	428	60.62	56.97	64.16
	Fometa	121	17.14	14.54	20.09
	Siteu	157	22.24	19.33	25.45
**Study level**	No education	257	36.40	32.94	40.02
	Primary level	83	11.76	9.58	14.34
	Secondary level	132	18.70	15.99	21.74
	University level	234	33.14	29.77	36.70
**Occupation**	Trader	103	14.59	12.18	17.38
	Private sector employee	182	25.78	22.69	29.13
	Public sector employee	189	26.77	23.64	30.16
	Student	119	16.86	14.28	19.79
	Unemployed	113	16.01	13.49	18.89
**Marital status**	Single	219	31.02	27,.72	34.53
	Married	225	31.87	28.54	35.40
	Widow(er)	262	37.11	33.63	40.73

CI: confidence interval

**Table 2:** patient’s history and comorbidities

Variables	Terms	Number (n)	Percentage (%)	CI (95%)	
**Hypertensive siblings**	Don’t Know	62	8.87	6.98	11.21
	No	463	66.24	62.65	69.65
	Yes	174	24.89	21.83	28.23
	**Total**	**699**	**100**		
**Hypertensive mother**	Don’t Know	67	9.7	7.71	12.13
	No	460	66.57	62.97	69.99
	Yes	164	23.73	20.71	27.05
	**Total**	**691**	**100**		
**Hypertensive father**	Don’t Know	110	15.58	13.09	18.44
	No	487	68.98	65.47	72.28
	Yes	109	15.44	12.96	18.29
	**Total**	**706**	**100**		
**Presence of comorbidity or cardiovascular risk factor**	No	159	23.04	20.06	26.33
	Yes	531	76.96	73.67	79.94
	**Total**	**690**	**100**		
**Alcohol consumption**	No	531	75.21	71.90	78.26
	Yes	175	24.79	21.74	28.10
	**Total**	**706**	**100**		
**Active smoking**	No	601	85.13	82.31	87.56
	Yes	105	14.87	12.44	17.69
	**Total**	**706**	**100**		
**Diabetes**	No	605	85.69	82.92	88.08
	Yes	101	14.31	11.92	17.08
	**Total**	**706**	**100**		
**Gout**	No	667	94.48	92.54	95.93
	Yes	39	5.52	4.07	7.46
	**Total**	**706**	**100,00**		
**Known hypertension**	No	560	79.32	76.18	82.15
	Yes	146	20.68	17.85	23.82
	No	**706**	**100**		
**Obesity**	No	676	95.75	94	97.01
	Yes	30	4.25	2.99	6.00
	**Total**	**706**	**100**		
**Sedentary life>**	No	654	92.63	90.47	94.34
	Yes	52	7.37	5.66	9.53
	**Total**	**706**	**100**		

CI: confidence interval

**Table 3:** patient’s complaints distribution

Variables	Number	Percentage %	CI (95%)	
**Participants’ complaints**				
Anxiety	33	4.67	0.30	1.65
Asthenia	189	26.77	5.20	9.01
Reduced sight	105	14.87	5.41	9.22
Headaches	108	15.30	6	11
Dyspnea	95	13.46	2.41	5.18
Hemiplegia	7	0.99	0.48	2.04
Palpitations	88	12.46	1.62	3.99
Dizziness	81	11.47	2.41	5.18

CI: Confidence interval

**Figure 3:**
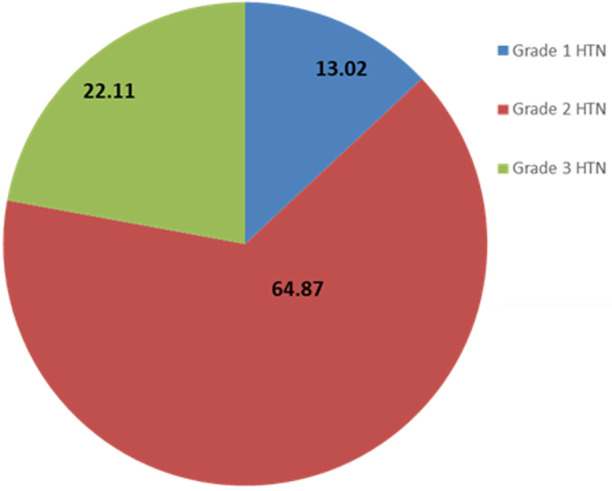
hypertension (HTN) classification among the study population

**Hypertension prevalence and classification:** hypertension prevalence was 57.6% (407 participants) among the study population. It was classified into grade 1 (53 participants, 13.02%), grade 2 (264 participants, 64.87%), and grade 3 (90 participants, 22.11%). Figure 3 presents the hypertension classification. Hypertension was more frequent among women without significance. However, people with hypertension were older than those without (55.3 VS 50.2 years, p<0.001).

**Factors associated with hypertension:** in multivariate analysis, the factors associated with hypertension were: age over 40 years (aOR: 3.23, 95% CI 1.91 - 5.47; p<0.001), being a public worker (aOR: 4.82, 95% CI 2.44 - 9.54; p <0.001), being unemployed (aOR: 2.95, 95% CI 1.32 - 6.57; p=0.008), tobacco consumption (aOR: 1.93, 95% CI 1.49 - 2.38; p<0.001), hypertension among siblings (aOR: 4.56, 95% CI 1.30 - 15.95; p=0.017), diabetes mellitus (aOR: 3.09, 95% CI 1.61 - 5.94; p<0.001), obesity (aOR: 2.8, 95 CI 1.10 - 7.74; p<0.001), previous education (over salt consumption: [aOR: 1.7 95% CI 1.08 - 2.67; p<0.001], alcohol consumption: [aOR: 2.12, 95% CI 1.24 - 3.61; p<0.04] and weight loss [aOR: 2.51, 95% CI 1.76 - 3.57; p<0.001]), presence of palpitations (aOR: 22.63, 95% CI 1.83 - 278.6; p=0.015]) and reduced vision (aOR: 1.97, 95% CI 1.08 - 3.6; p=0.026). Table 4 and Table 4.1 present the multivariate analysis.

**Table 4:** factors associated with hypertension in multivariate analysis

Variables	Terms	All (n)	HTN (yes)	HTN (No)	cOR (95% CI)	cP-value	aOr (95% CI)	aP-value
**Age group (years)**	< 40	177	62 (16.0)	115 (36.1)				
	≥ 40	529	325 (84.0)	204 (63.9)	2.96 (1.32-4.18)	<0.001	3.23 (1.91-5.47)	<0.001
**Sex**	Female	445	247 (63.8)	198 (62.1)	1.08 (0.79-1.46)	0.631		
	Male	261	140 (36.2)	121 (37.9)				
**Health areas**	Fiala Foreke	428	232 (59.9)	196 (61.4)	0.94 (0.69-1.27)	0.686		
	Fometa	121	71 (18.3)	50 (15.7)	1.21 (0.81-1.80)	0.348		
	Siteu	157	84 (21.7)	73 (22.9)	0.93 (0.65-1.33)	0.708		
**Study level**	No education	257	152 (39.3)	105 (32.9)	1.32 (0.97-1.80)	0.080		
	Primary level	83	43 (11.1)	40 (12.5)	0.87 (0.55-1.38)	0.558		
	Secondary level	132	74 (18.3)	58 (18.2)	1.06 (0.73-1.56)	0.750		
	University level	234	84 (21.4)	116 (36.4)	0.77 (0.56-1.05)	0.099		
**Occupation**	Trader	103	60 (15.5)	43 (13.5)	1.18 (0.77-1.80)	0.448		
	Private sector employee	182	90 (23.3)	92 (28.8)	0.75 (0.53-1.048)	0.091		
	Civil servant	189	118 (30.5)	71 (22.3)	1.53 (1.09-2.15)	0.014	4.84 (2.44-9.54)	<0.001
	Student	119	43 (11.1)	75 (23.8)	0.40 (0.27-0.60)	<0.001		
	Unemployed	113	76 (19.6)	37 (11.6)	1.86 (1.22-2.85)	0.004	2.95 (1.32-6.57)	0.008
**Marital status**	Single	219	100 (25.8)	119 (37.3)	0.59 (0.42-0.81)	0.001		
	Married	225	135 (34.9)	90 (28.2)	1.36 (0.99-1.88)	0.058		
	Widow(er)	262	152 (39.3)	110 (34.5)	1.23 (0.90-1.67)	0.189		
**Hypertensive siblings**	Don’t Know	62	41 (10.8)	21 (6.6)	1.70 (0.98-2.95)	0.054		
	No	463	177 (46.5)	286 (89.9)	0.09 (0.06-0.15)	<0.001		
	Yes	174	163 (42.8)	11 (3.5)	20.87 (11.06-39.37)	<0.001	4.56 (1.30-15.95)	0.018
	Total	699	381 (100)	318 (100)				
**Hypertensive mother**	Don’t Know	67	44(11.8)	23 (7.2)	1.73 (1.02-2.93)	0.041		
	No	460	207(55.6)	253(79.3)	0.33 (0.23-0.46)	<0.001		
	Yes	164	121 (32.5)	43 (13.5)	3.09 (2.10-4.56)	<0.001		
	Total	691	372 (100)	379 (100)				
**Hypertensive father**	Don’t Know	110	74 (19.1)	36 (11.3)	1.86 (1.21-2.86)	0.004		
	No	487	268 (69.3)	219 (68.7)	1.03 (0.75-1.42)	0.864		
	Yes	109	45 (11.6)	64 (20.1)	0.52 (0.35-0.79)	0.002		
	Total	706	387 (100)	319 (100)				
**Presence of comorbidity or cardiovascular risk factor**	No	159	300 (80.9)	231(72.4)	1.61 (1.13-2.30)	0.009		
	Yes	531	71 (19.1)	88 (27.6)				
	Total	690	371 (100)	319 (100)				
**Alcohol consumption**	Yes	175	91 (23.5)	84 (26.3)	0.86 (0.61-1.21)	0.388		
	No	531	296 (76.5)	235 (73.7)				
	Total	706	387 (100)	319 (100)				
**Active smoking**	Yes	105	68 (17.6)	37 (11.6)	1.62 (1.06-2.50)	0.026		
	No	601	319 (82.4)	282 (88.4)				
	Total	706	387 (100)	319(100)				
**Diabetes**	Yes	101	90 (23.3)	11 (3.4)	8.84 (4.45-16.19)	<0.001	3.09 (1.61-5.94)	<0.001
	No	605	297 (76.7)	308 (96.6)				
	Total	706	387 (100)	319 (100)				
**Gout**	Yes	39	39 (10.1)	0	NA	<0.001		
	No	667	348 (89.9)	319 (100)				
	Total	706	387 (100)	319 (100)				
**Known Hypertension**	Yes	146	118 (30.5)	28 (8.8)	4.56 (2.92-7.11)	<0.001		
	No	560	269 (69.5)	291 (91.2)				
	Total	706	387 (100)	319 (100)				
**Obesity**	Yes	30	27 (7.0)	3 (0.9)	7.90 (2.37-26.29)	<0.001	2.8 (1.1-7.74)	0.011
	No	676	360 (93.0)	316 (99.1)				
	Total	706	387 (100)	319 (100)				
**Sedentary life>**	Yes	52	49 (12.7)	3 (0.9)	15.27 (4.71-49.48)	<0.001		
	No	654	338 (87.3)	316 (99.1)				
	Total	706	387 (100)	319 (100)				

CI: confidence interval; HTN: hypertension; cOR: crude odd ratio; aOR: adjusted odd ratio, cP-value: crude p-value; aP-value: adjusted p-value

**Table 4.1:** factors associated with hypertension in multivariate analysis

Variables	Terms	All (n)	HTN (yes)	HTN (No)	cOR (95% CI)	cP-value	aOr (95% CI)	aP-value
**Education on weight reduction**	Yes	190	140 (36.2)	50 (15.7)	3.05 (2.11-4.39)	<0.001	2.51 (1.76-3.57)	<0.001
	No	516	247 (63.8)	269 (84.3)				
	Total	706	387 (100)	319 (100)				
**Education on salt consumption**	Yes	220	182 (47.0)	38 (11.9)	6.56 (4.43-9.73)	<0.001	1.70 (1.08-2.67)	0.02
	No	486	205 (53.0)	281 (88.1)				
	Total	706	387 (100)	319 (100)				
**Education on alcohol consumption**	Yes	219	151 (39.0)	68 (21.3)	2.36 (1.69-3.31)	<0.001	2.12 (1.24-361)	0.005
	No	487	236 (61.0)	251 (78.7)				
	Total	706	387 (100)	319 (100)				
**Education on tobacco consumption**	Yes	181	145 (37.5)	36 (11.3)	4.71 (3.15-7.05)	<0.001		
	No	525	242 (62.5)	283 (88.7)				
	Total	706	387 (100)	319 (100)				
**Education on Physical activity**	Yes	295	213 (55.0)	82 (25.7)	3.36 (2.57-4.88)	<0.001		
	No	411	174 (45.0)	237 (74.3)				
	Total	706	387 (100)	319 (100)				
**Anxiety**	Yes	5	5 (1.6)	0	NA	0.067		
	No	701	382 (98.7)	319 (100)				
	total	706	387 (100)	319 (100)				
**Asthenia**	Yes	47	44 (11.4)	3 (0.9)	13.51 (4.15-43.95)	<0.001		
	No	659	343 (88.6)	316 (99.1)				
	Total	706	387 (100)	319 (100)				
**Reduced vision**	Yes	50	45 (11.6)	5 (1.6)	8.26 (3.24-21.08)	<0.001	1.97 (1.08-3.60)	0.026
	No	656	342 (88.4)	314 (98.4)				
	Total	706	387 (100)	319 (100)				
**Headaches**	Yes	5	0	5 (1.6)	NA	0.019		
	No	701	387 (100)	314 (98.4)				
	Total	706	387 (100)	319(100)				
**Dyspnea**	Yes	25	22 (5.7)	3 (0.9)	6.35 (1.88-21.41)	0.001		
	No	681	365 (94.3)	316 (99.1)				
	Total	706	387 (100)	319 (100)				
**Hemiplegia**	Yes	26	26 (6.7)	0	NA	<0.001		
	No	680	361 (93.3)	319 (100)				
	Total	706	387 (100)	319 (100)				
**Palpitations**	Yes	18	18 (4.7)	2 (0.6)	7.73 (1.16 - 9.0)	<0.001	22.63 (1.83-278.6)	0.015
	No	688	369 (95.3)	317 (99.4)				
	Total	706	387 (100)	319 (100)				
**Dizziness**	Yes	25	22 (5.7)	3 (0.9)	6.35 (1.88-21.41)	0.001		
	No	681	365 (94.3)	316 (99.1)				
	Total	706	387 (100)	319 (100)				

CI: confidence interval; HTN: hypertension; cOR: crude odd ratio; aOR: adjusted odd ratio, cP-value: crude p-value; aP-value: adjusted p-value

## Discussion

This study aimed to determine the prevalence of hypertension in people living in the Dschang Health District and identify their risk factors. This study found a high prevalence of hypertension in these participants and allowed us to identify several risk factors such as age group above 40 years, being a civil servant, being unemployed, having a sibling hypertensive, being diabetic, being obese, being an active smoker, having an increased BMI, previous education on salt, previous education on alcohol consumption, presence of palpitations and sight reduction. The prevalence of hypertension in our study was 57.6%. This was higher than previous findings (40%) in a rural population [23]. Another study, in the Democratic Republic of Congo, found a prevalence of 32.52%, similar to the prevalence found among a group of prisoners at the central prison of Yaoundé (39.6%) [24,25]. Our findings were however similar to those from Burkina Faso, where they found, in a hospital study a prevalence of around 65% [26]. The difference in prevalence between our study and the studies above could be explained by several parameters: a rural population (older than the urban one), a greater prevalence within this population of participants already known to be hypertensive and a high prevalence of other cardiovascular risk factors.

Participants with grade 3 hypertension were more common in Burkina Faso's study followed by grade 2 at the end of grade 1, and people on treatment were only 15% [17]. In this study, the most represented grades were grade 2, then grade 3 followed by grade 1. Participants aware of living with hypertension accounted for around 20% of the study population (35.57% of all people with hypertension). This synoptic difference could be explained by several things, namely the place of recruitment here we recruit in the community while for Salam *et al*. the recruitment took place in hospital service [26]. Secondly, many of our participants already had either a diagnosis of hypertension or education on tobacco, alcohol, or weight loss.

In this study population, the determinants of hypertension in multivariate analysis were: i) socio-demographic characteristics: the age group between 40 and 96 years, the profession of public sector employees, and the unemployed were correlated with hypertension. This is in line with the studies conducted in Cameroon, in the Democratic Republic of the Congo and in Burkina regarding age [24,26,27]. But it should be noted in Cameroon, this was the age group over 70. Married and widowed status were correlated with arterial hypertension but this association was not strong and not significant. This is contradictory with studies conducted in India, Ecuador, and Brazil where they have shown the correlation between these statuses and high blood pressure [28-30]; ii) family history of high blood pressure: having a hypertensive sibling was also associated with an increased risk of high blood pressure, unlike the presence of high blood pressure in the mother. This is in line with the study in the Democratic Republic of Congo where participants with a history of hypertension in the siblings have where participants with a history of hypertension in the siblings were more likely to have arterial hypertension [20]; iii) clinical characteristics: being diabetic, obese, or an active smoker correlated with an increased risk of hypertension in our study. This has already been demonstrated in the Democratic Republic of Congo concerning obesity [24]. This is consistent with data from Oliveira *et al*. in Portugal [31].

Regarding the functional signs found in our participants, it should be noted that only palpitations and decreased vision were associated with the presence of hypertension. This contradicts the findings of El Kardoudi *et al*. in Morocco where dyspnea, chest pain, and decreased vision as the main functional signs associated with arterial hypertension [32]. The difference is probably because this study was conducted once again in a University Hospital Centre and with a much more educated population. In the study at the Yaoundé Central Hospital in Cameroon, the functional signs associated with hypertension were, in descending order, loss of vision; chest pain, and hemiplegia [27]. This is always explained by the place of the study indeed it is very difficult to find in the community a person who has had a stroke or a person complaining of typical chest pain.

Hypertension should be checked on every adult coming in consultation particularly those aged above 40 years, civil servant or unemployed, with a familial history of hypertension, known obese or smoker, or living with diabetes. The presence of functional signs such as palpitations or decreased vision should also prompt the search for hypertension. This cross-sectional study should be interpreted in light of some limitations. Although this is one of the most important community studies in Cameroon, we carried out this study in only one Health District with urban and rural populations, findings cannot be extrapolated to others. The other limitations were the fact that individuals with hypertension have not undergone the most adequate measurement method to confirm hypertensive status.

## Conclusion

Almost 6 over 10 adults in the Dschang Health District are hypertensive. Grade 2 hypertension followed by grade 3 was the most represented. Factors associated with hypertension in this population were: age over 40 years, being a civil servant, being unemployed, being diabetic, being obese, previous education on salt and alcohol consumption, and the presence of palpitations or reduced vision. There is a need to implement hypertension prevention strategies in urban and semi-urban settings.

### What is known about this topic

- *The national prevalence of hypertension is well established, but its prevalence in semi-urban areas is poorly known*.

### What this study adds

- *A high prevalence of hypertension was found in semi-urban areas (Dschang Health District); the main risk factors identified were alcohol and tobacco consumption, being a civil servant or unemployed, hypertension among siblings, smoking, diabetes, physical inactivity, and obesity, the presence of palpitations, and reduced vision;*
- *How this study might affect research, practice, or policy: there is a need to implement hypertension prevention strategies in semi-urban and rural settings similar to urban areas*.
